# Research Progress in the Molecular Mechanisms, Therapeutic Targets, and Drug Development of Idiopathic Pulmonary Fibrosis

**DOI:** 10.3389/fphar.2022.963054

**Published:** 2022-07-21

**Authors:** Hongbo Ma, Xuyi Wu, Yi Li, Yong Xia

**Affiliations:** ^1^ Department of Rehabilitation Medicine, State Key Laboratory of Biotherapy and Cancer Center, National Clinical Research Center for Geriatrics, West China Hospital, Sichuan University, Chengdu, China; ^2^ West China School of Pharmacy, Sichuan University, Chengdu, China; ^3^ Key Laboratory of Rehabilitation Medicine in Sichuan Province/Rehabilitation Medicine Research Institute, Chengdu, China

**Keywords:** idiopathic pulmonary fibrosis, cells crosstalk, gene mutations, epigenetics, emerging drugs

## Abstract

Idiopathic pulmonary fibrosis (IPF) is a fatal interstitial lung disease. Recent studies have identified the key role of crosstalk between dysregulated epithelial cells, mesenchymal, immune, and endothelial cells in IPF. In addition, genetic mutations and environmental factors (e.g., smoking) have also been associated with the development of IPF. With the recent development of sequencing technology, epigenetics, as an intermediate link between gene expression and environmental impacts, has also been reported to be implicated in pulmonary fibrosis. Although the etiology of IPF is unknown, many novel therapeutic targets and agents have emerged from clinical trials for IPF treatment in the past years, and the successful launch of pirfenidone and nintedanib has demonstrated the promising future of anti-IPF therapy. Therefore, we aimed to gain an in-depth understanding of the underlying molecular mechanisms and pathogenic factors of IPF, which would be helpful for the diagnosis of IPF, the development of anti-fibrotic drugs, and improving the prognosis of patients with IPF. In this study, we summarized the pathogenic mechanism, therapeutic targets and clinical trials from the perspective of multiple cell types, gene mutations, epigenetic and environmental factors.

## 1 Introduction

Idiopathic pulmonary fibrosis (IPF) is a progressive, life-threatening, interstitial lung disease of unknown pathogenesis. IPF has a familial and sporadic onset with a poor prognosis, and death usually occurs within 2–5 years of diagnosis due to secondary respiratory failure (Noble et al., 2012). CT imaging of IPF usually shows a typical usual interstitial pneumonia (UIP) pattern, characterized by irregular reticular opacities with obligatory honeycombing, associated with traction bronchiectasis. IPF also exhibits histological features of UIP/IPF pattern characterized by architecture remodeling due to dense fibrosis with frequent honeycombing, patchy lung involvement by fibrosis, subpleural and/or paraseptal distribution, fibroblast foci at the edge of dense scars (Spagnolo et al., 2018; Baratella et al., 2021). Although the etiology of IPF is unknown, various imbalances centered on alveolar epithelial cell/fibroblast apoptosis imbalance has been shown to play an important role in the pathogenesis of IPF (Wang et al., 2021). Therefore, it is necessary to understand the respective roles and interactions of alveolar epithelial cells, fibroblasts, immune cells, and extracellular matrix (ECM) in the complex crosstalk. In addition, we discuss potential factors affecting these pro-fibrotic cells, including genetic mutations, epigenetic alterations, environmental factors and aging, with the aim of finding the underlying cause of the disease. The currently approved IPF treatment drugs are pirfenidone and nintedanib, both of which can slow the progression of IPF, but there is no evidence that they can reverse IPF-related pulmonary fibrosis (Chu et al., 2020). Lung transplantation is the only option for patients with end-stage IPF (Lederer and Martinez, 2018; Villavicencio et al., 2018). Therefore, there is a necessity to develop novel agents for the treatment of IPF. This article reviews the roles of various cells and extracellular matrix associated with pathogenic mechanisms, potential pathogenic factors, and the latest information on clinical trials of IPF.

## 2 The Pathological Process of Idiopathic Pulmonary Fibrosis

The current paradigm suggests that IPF occurs as a result of epithelial injury and dysregulation of the epithelial/mesenchymal crosstalk, which continuously activates multiple interconnected downstream profibrotic pathways, ultimately leading to an abnormal repair response and decreased lung function (Spagnolo et al., 2021).

**FIGURE 1:**
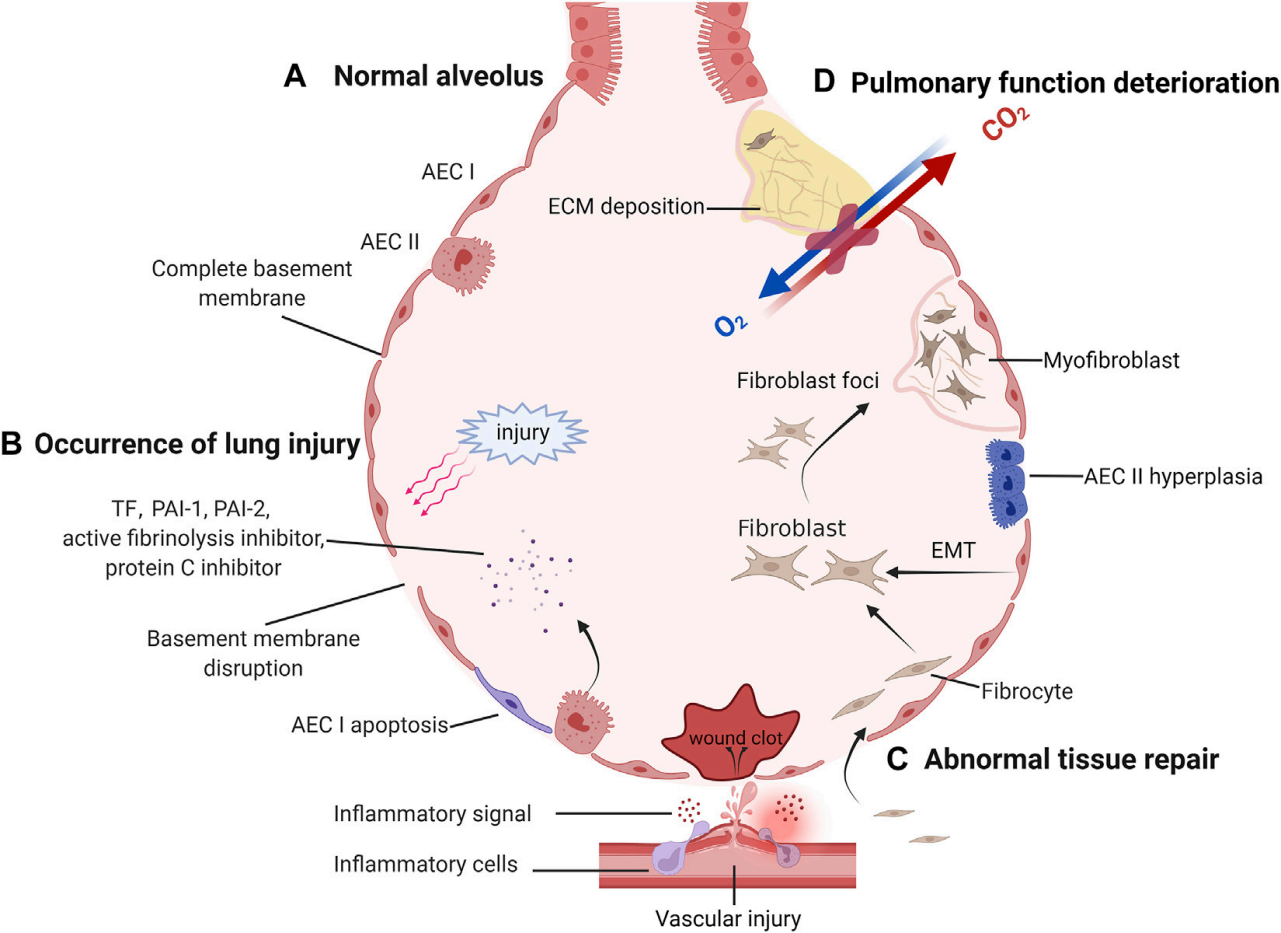
The pathological process of idiopathic pulmonary fibrosis. **(A)** After normal alveoli are damaged and abnormally repaired, irreversible lung function deterioration occurs. Normal alveolus has a complete basement membrane and gas exchange function. **(B)** When the basement membrane continuity is disrupted by external injury, the damaged capillaries and activated AECs release inflammatory signals and coagulation factors, forming a local inflammatory microenvironment. **(C)** If the damage persists, abnormal repair will be initiated. Lung mesenchymal progenitors, fibrocytes recruited to the lung, and endothelial cells undergoing EMT can aggregate to form fibroblasts foci and differentiate into matrix-secreting myofibroblasts. To compensate for the local blood supply to the alveoli, new blood vessels are gradually formed. **(D)** As fibroblast foci increased, more ECM was deposited and cross-linked together, triggering a deterioration in lung compliance and gas exchange function.

In the next sections, we provide a brief overview of the pathogenic mechanisms of IPF (Figure 1). Damage to alveolar epithelial cells in response to external stimuli leads to disruption of the basement membrane and release of large amounts of cytokines. Many cytokines (interleukins, chemokines, and growth factors) are released by alveolar epithelial cells (AEC) to recruit and activate inflammatory cells and fibroblasts (Phan et al., 2021; She et al., 2021). Coagulation factors (tissue factor (TF), plasminogen activation inhibitors (PAI 1 and PAI 2), fibrinolysis inhibitors and protein C inhibitors) induce a microenvironment that promotes coagulation and inhibits fiber degradation (King et al., 2011; Selman and Pardo, 2014; Betensley et al., 2016). In addition to microenvironmental changes in the lung, cellular processes (apoptosis, senescence, epithelial-mesenchymal transition, endothelial-mesenchymal transition, and epithelial cell migration) have been shown to play a key role in IPF-associated tissue remodeling (Phan et al., 2021). In addition, fibroblasts differentiate into myofibroblasts and secrete large amounts of extracellular matrix, which eventually leads to the formation of fibroblast foci and the development of pulmonary fibrosis (Yagihashi et al., 2016). In conclusion, following injury to the AEC, the lung microenvironment and cellular processes are altered, which initiates abnormal repair and ultimately leads to the development of IPF and pulmonary function deterioration.

## 3 Important Cells and Extracellular Matrix Involved in the Pathogenesis of IPF

There are AECs, alveolar capillary endothelial cells, immune cells, fibroblasts, and mesenchymal progenitor cells near the alveoli. These cells maintain the homeostasis of the alveolar environment under normal physiological conditions. However, in the pathophysiological process of IPF, the intercellular crosstalk leads to reprogramming of cell phenotype. AECs, endothelial cells, and immune cells work together through multiple signaling pathways to regulate fibroblast phenotype. Then, fibroblasts recruitment, proliferation, differentiation, and secretion of extracellular matrix directly lead to fibrosis and exhaustion of pulmonary function. therefore, it is necessary to clarify the roles of various cells and extracellular matrix in the development of IPF.

### 3.1 Alveolar Epithelial Cells and Endothelial Cells

**FIGURE 2:**
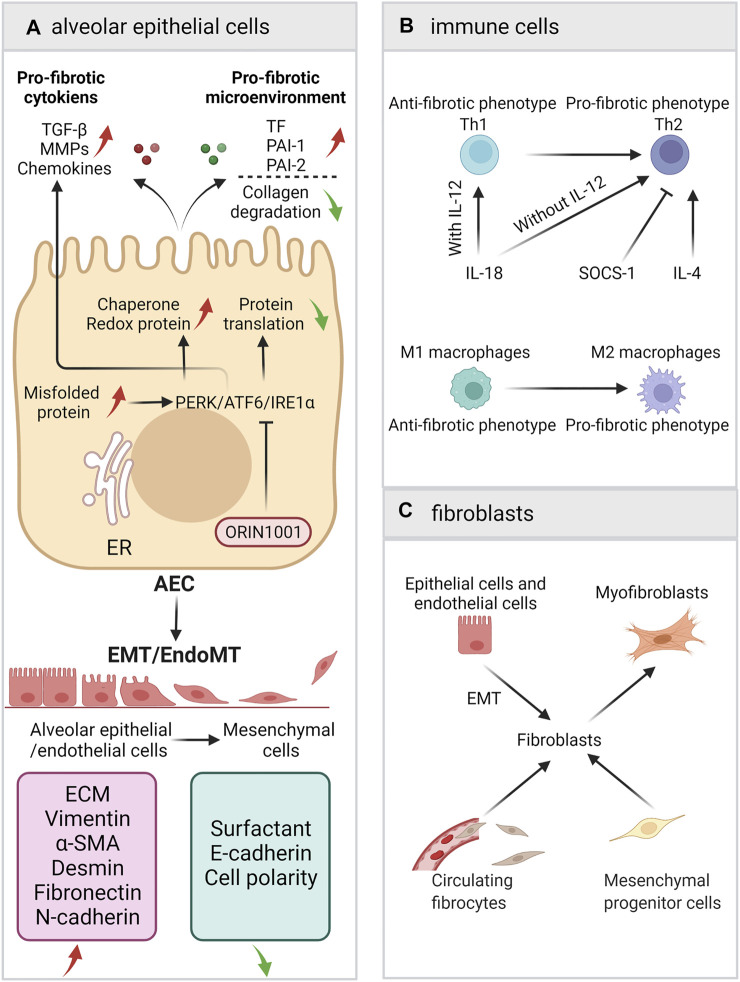
A schematic view of the roles of AECs, immune cells and fibroblasts. **(A)** Alveolar epithelial cells/endothelial cells participate in IPF via ER stress/UPR, EMT, coagulation cascade, and the secretion of a variety of signaling factors. **(B)** The balance of Th1/Th2 and M1/M2. **(C)** The sources of fibroblasts and the fibroblast-to-myofibroblasts differentiation.

Alveolar epithelial cells/endothelial cells participate in IPF via various ways, including unfolded protein response (UPR), Epithelial-mesenchymal transition (EMT), coagulation cascade, angiogenesis, and the secretion of a variety of signaling factors (such as TGF-β) (Margaritopoulos et al., 2017; Upagupta et al., 2018; Chanda et al., 2019; Hill et al., 2019; Salton et al., 2019; Selman and Pardo, 2020). In this section, we show how AECs are involved in pulmonary fibrosis (Figure 2A).

#### 3.1.1 Unfolded Protein Response

The UPR of AEC is one of the underlying mechanisms for the development of IPF. As an important site for cellular protein synthesis, the ER (endoplasmic reticulum) must maintain relative homeostasis. Under pathological conditions [e.g., viral infection, smoking, asbestos exposure, ROS, hypoxia, senescence, mechanical stretching, proteasome dysfunction, and autophagy disorders (Burman et al., 2018)], misfolded proteins accumulate in the ER of AECs, resulting in ER stress. To restore protein metabolism homeostasis, PERK/ATF6/IRE1α (Protein kinase R-like endoplasmic reticulum kinase/activating transcription factor 6/inositol-requiring enzyme 1α) receptors will be activated, followed by the activation of the UPR to reduce overall protein translation and increase the expression of chaperone and redox proteins. The UPR/ER stress regulates AEC apoptosis and EMT (Burman et al., 2018). However, UPR can also participate in pulmonary fibrosis by increasing the expression of profibrotic mediators, such as TGF-β1, platelet-derived growth factor (PDGF), CXCL12, and CCL2 (Zolak and de Andrade, 2012; Wolters et al., 2014). Because the upstream targets of the UPR/ER stress are of great significance for the maintenance of cell survival and organ development, pathways targeting upstream molecules may cause severe cytotoxicity. Therefore, targeting downstream molecular pathways or chaperones seems to be a better choice. (Burman et al., 2018). Currently, ORIN1001, which targets inositol-requiring transmembrane kinase endoribonuclease-1α (IRE1α), is undergoing a phase I clinical trial for the treatment of IPF(NCT04643769). The crosstalk between autophagy and endoplasmic reticulum stress is important in pulmonary fibrosis (Ghavami et al., 2018; Maciel et al., 2018). Therapeutic approaches targeting autophagy have been shown to have great potential in cancer and aging (Xia et al., 2021; Cassidy and Narita, 2022). Interestingly, studies have found that autophagy also plays an important role in IPF. TGF-β can inhibit autophagy in fibroblasts, while rapamycin and Tubastatin can promote autophagy and inhibit bleomycin-mediated pulmonary fibrosis (Patel et al., 2012; Saito et al., 2017).

#### 3.1.2 Epithelial-Mesenchymal Transition

EMT refers to a process in which AECs gradually lose their epithelial characteristics under specific stimuli and conditions, with the subsequent appearance of interstitial cells characteristics. During this process, AECs are reprogrammed with changes in secretory phenotype, cytoskeletal proteins, intercellular junctions, and cell polarity (Salton et al., 2019). The conditions that induce EMT in AECs include alveolar epithelial cell injury and abnormal apoptosis, the UPR/ER stress, mechanical stress, smoking, and infection (Salton et al., 2019). Under the repeated stimuli, AECs are severely damaged and cannot complete repair processes and re-epithelialization normally, resulting in reprogramming and manifesting as abnormal repair processes (Salton et al., 2020). TGF-β, epidermal growth factor (EGF), fibroblast growth factor (FGF), IL-1, connective tissue growth factor factors (CTGF), insulin-like growth factor-2 (IGF-2), nuclear factor-kb (NF-kB) and Wnt can activate the transcription factors SNAIL, TWIST1 and ZEB through a variety of cytokine pathways to directly initiate EMT (Salton et al., 2019). FGFR1-3 inhibitor SKLB-YTH-60 ameliorates EMT and fibrosis in bleomycin-induced lung fibrosis mouse models (Liu et al., 2021).

However, in recent years, the central role of EMT in the pathogenesis of IPF has been questioned. The localization of type 2 epithelial cells by markers showed that the conversion of epithelial cells into myofibroblasts was incomplete (Gjorevski et al., 2012) and that the marker protein of myofibroblasts, α-SMA, and EMT epithelial cells could not be colocalized, indicating that epithelial cells may not completely convert to fibroblasts (Rock et al., 2011); additionally, mesenchymal AECs have a very limited ability to secrete ECM (Yao et al., 2019). Studies have shown that EMT indirectly promotes the formation of a profibrotic microenvironment through the dysregulation of paracrine signals between epithelial cells and mesenchymal cells (Hill et al., 2019; Yao et al., 2019). Therefore, although there is a large amount of evidence that EMT does exist in IPF, due to the latest lineage tracing results, EMT is more regarded as an indirect process. Nevertheless, the profibrotic microenvironment that regulates the occurrence of EMT is still quite promising in the inhibition of IPF (Hill et al., 2019).

#### 3.1.3 Coagulation Cascade and Angiogenesis

Under pathological conditions, the coagulation cascade and angiogenesis are important driving forces for the promotion of pulmonary fibrosis. Because tissue factor can activate PAI 1, PAI 2, fibrinolysis inhibitors and protein C inhibitors through the coagulation cascade, resulting in a local pro-coagulation microenvironment, it inhibits the degradation of the ECM in this microenvironment and promotes the differentiation of fibrocytes (King et al., 2011; Selman and Pardo, 2014; Betensley et al., 2016). IPF patients have a relatively lower number of endothelial progenitor cells, which may potentially contribute to suppressed repair of the damaged pulmonary endothelium and thereby may drive the sequence of events in profibrogenic direction (Malli et al., 2013). Besides, it is reported that compensatory pro-angiogenic VEGF increases, which is a pro-fibrotic mediator (Malli et al., 2013).

#### 3.1.4 Pro-Fibrotic Secretory Phenotype of AEC

In addition to the above pathways, AECs also participate in pulmonary fibrosis through the secretion of a variety of mediators, including growth factor (TGF-β, PDGF/CTGF/IGF-I/insulin-like growth factor binding proteins 3 and 5), matrix metalloproteinases (MMP1/MMP2/MMP7), chemokines (CCL17/CCL2/CXCL12), pigment epithelium-derived factor, autotaxin, sphingosine-1-phosphate, neuregulin (NRG) 1α, growth and differentiation factor 15 (GDF15), transmembrane protease serine 4 (TMPRSS4), tumor necrosis factor-alpha (TNF-α), osteopontin, and angiotensinogen. Mareike Lehmann et al. summarized the possible roles of these cytokines in detail in the occurrence and development of IPF (Selman and Pardo, 2020).

### 3.2 Immune Cells

A large number of studies have demonstrated that immune cells play a role in IPF. However, anti-inflammatory therapies, i.e., TNF-α monoclonal antibodies (Utz et al., 2003; Raghu et al., 2008) and glucocorticoids (Raghu et al., 2012), fail to achieve primary outcomes in clinical trials. Neither of these promising conventional treatments prevent a decline in forced vital capacity (FVC) or the progression of pulmonary fibrosis or benefit survival. In addition, a phase 3, randomized, double-blind, placebo-controlled study including 826 participants (NCT00075998) showed that the subcutaneous injection of IFNγ-1b neither improved the FVC of patients nor prolonged the survival time of patients. These clinical trials are important milestones for clinical treatment strategies for IPF, causing a shift from traditional anti-inflammatory treatment to simultaneous interventions for multiple pathogenic links. Past treatments generally treated inflammation as a whole while ignoring the dual profibrotic/antifibrotic roles of different inflammatory factors and different inflammatory cells. Perhaps by further distinguishing the roles of different inflammatory factors and pathways involved in different inflammatory cells, more precise therapeutic targets can be found. Therefore, inflammation, as one of the potential pathogeneses of IPF, remains an important focus of research.

With the deepening of research in the field of immunization, the role of immune factors in IPF has received increasing attention. Although the diagnostic criteria for IPF require the exclusion of autoimmune diseases as the underlying pathogenesis, many IPF patients still have unexplained elevated autoantibodies, and some autoantibodies are associated with acute exacerbations of IPF (AE-IPF) (Ogushi et al., 2001; Kurosu et al., 2008; Taillé et al., 2011; Kahloon et al., 2013). To inhibit the function of B cells, Ianalumab (B-cell activating factor receptor mAb) and Rituximab (CD20 mAb) have entered phase II clinical trials.

Heukels conducted a detailed review on the role of each immune cell in IPF and the correlation of each cell type with the development of fibrosis (Heukels et al., 2019). Nevertheless, the mechanism of action of some immune cells in the pathogenesis of IPF is still not very clear. The following subsections introduce the roles of the Th1/Th2 balance and the M1/M2 balance in IPF, although these paradigms simplify the role of immune cells (Figure 2B).

#### 3.2.1 Th1/Th2 Balance

Th1 (helper T cell type 1) cells are helper T cells produced by CD4^+^ cells under IFN-γ/IL-12 induction. CXC chemokine receptor 3 expressed by Th1 cells can recognize interferon-inducible T cell a chemoattractant (I-TAC), interferon g-inducible protein of 10 kD (IP-10), and monokine induced by interferon gamma (Mig) (Sumida et al., 2008). The main function of Th1 is to secrete IFN-γ. IFN-γ is considered to be antifibrotic and can reduce the production of ECM (Bouros et al., 2006; Smaldone, 2018).

Th2 (helper T cell type 2) are helper T cells produced by CD4^+^ cells under IL-4 induction. Th2 cells that express CC chemokine receptor 4 can recognize thymus- and activation-regulated chemokines (TARCs) and macrophage-derived chemokines (MDCs) (Sumida et al., 2008). The main function of Th2 cells is to secrete IL-4/IL-5/IL-13; these interleukins are considered to promote fibrosis (Romagnani, 2000).

Th2 polarization has been observed in IPF. In the BALF of IPF patients and in systemic circulation, the levels of Th1 cells and their secretion of IFN-γ are relatively low, and the levels of Th2 cells and their secretion of IL-4/IL-5/IL-13 are relatively high (Hams et al., 2014). In addition, in a bleomycin-induced mouse model, an increase in IFN-γ levels and a reduction in pulmonary fibrosis were observed after the administration of IL-12, an inducer of Th1 cells; in contrast, an increase in fibroblast proliferation and fibrosis was found with the use of IL-4, an inducer of Th2 cells (Hams et al., 2014; Passalacqua et al., 2017).

The inhibition of Th2 polarization is a possible direction for the treatment of IPF. Many factors can cause Th2 polarization. Galectin-1 and prostaglandin E2 promote Th2 polarization by inducing Th1 apoptosis and reducing the synthesis of Th1 inducers (Kaliński et al., 1997; Cedeno-Laurent and Dimitroff, 2012; Corapi et al., 2018), and suppressor of cytokine signaling-1 (SOCS-1) inhibits the expression of Th2 inducers to prevent excessive Th2 cell accumulation (Bao et al., 2014). Among the interleukins that regulate Th1 and Th2 differentiation, IL-18 is notable; the polarization direction mediated by IL-18 is regulated by IL-12. Under the synergistic effect of IL-12, IL-18 induces Th1 cells to produce IFN-γ, IL-12, and GM-CSF and upregulate the expression of IL-2Rα to promote an inflammatory response. In contrast, in the absence of IL-12, IL-18 induces the production of Th2-related cytokines, such as IL-13/IL-4, by T cells, NK cells, basophil cells, and mast cells and promotes the differentiation of Th2 cells (Wawrocki et al., 2016). IL-4 is another important interleukin that promotes Th2 differentiation and is an important marker of type 2 immunity. Studies have shown that significant polymorphisms are found in the IL-4 promoter of IPF patients and that these polymorphisms are strongly associated with IPF (Vasakova et al., 2006). In addition to cytokine therapy, a study used the serum IFN-γ/IL-4 ratio to represent the Th1/Th2 balance to predict the development of IPF and found that the IFN-γ/IL-4 ratio was associated with symptoms, imaging changes, FEV1 (forced expiratory volume in one second), FVC (Forced vital capacity), TLC (total lung capacity), and 6-min walking distance in IPF patients and can predict IPF progression (Peng et al., 2013).

#### 3.2.2 M1/M2 Balance

In addition to the Th1/Th2 balance, the balance of macrophage subpopulations also plays an important role in the pathogenesis of IPF. Because cytokines secreted by Th1/Th2 cells greatly affect the differentiation of M1/M2 macrophages, Th1/Th2 cells and M1/M2 macrophages interact with each other to jointly shape the type 1 and type 2 immune microenvironments (Wang et al., 2021).

The main function of M1 (type I macrophages) is to respond to lipopolysaccharide (LPS), IL-1, and IL-6. They can secrete type 1 immune factors such as IL-12, induced nitric oxide synthase (iNOS), TNF-α, IL-1β, IL-23, IL-6, and CXCL10 (Liu G. et al., 2019), thereby playing a role in the early stage of inflammation. The main function of M2 (type II macrophages) is to respond to type 2 immune factors (IL-4, IL-10, and IL-13), glucocorticoids, and immune complexes. They can secrete cytokines that promote tissue repair, limit inflammation (Martinez et al., 2008; Zhang et al., 2018; Liu G. et al., 2019), participate in immune regulation, suppress immune responses, and remodel tissue. M2 macrophages can also be divided into M2a, M2b, and M2c macrophages based on induction conditions and functions (Martinez et al., 2008).

M2 polarization is one of the important links in the occurrence and development of pulmonary fibrosis. The M2-mediated type 2 immune response is an important component of pulmonary fibrosis. M2 macrophages provide an important microenvironment for pulmonary fibrosis by secreting profibrotic substances such as CCL18, IL-10, TIMP1 (tissue inhibitors of metalloproteinases 1), TGF-β, FGF, PDGFα, IGF1, and VEGF (Prasse et al., 2006; Heukels et al., 2019; Wang et al., 2021). Macrophages can not only secrete chemokines to recruit other cells (like CCL18), but also are attracted by chemokines themselves. CCL2 and CCL3 are important signaling molecules involved in monocyte/macrophage recruitment and help macrophages migrate to the lungs. Therefore, the antagonism of CCL2 and CCL3 may have an antifibrotic effect (Iyonaga et al., 1994; Suga et al., 1999; Deshmane et al., 2009).

### 3.3 Fibroblasts and Myofibroblasts

Fibrocytes are monocyte progenitor cells with differentiation potential derived from bone marrow; these cells can differentiate into adipocyte cells, chondrocytes, osteoblasts, and fibroblasts under the action of different tissue environments and humoral factors. In the *in vitro* environment, CD45+/CD34+ primary bone marrow fibrocytes were stimulated by the ECM of IPF patients; then the hematopoietic surface antigens CD45 and CD34 rapidly disappeared, and mesenchymal markers, i.e., α-SMA, rapidly increased. The fibrocytes transformed into fibroblasts with a contractility like that of smooth muscle cells and a strong ability to synthesize ECM (Mori et al., 2005; Lama and Phan, 2006; Kage and Borok, 2012; Chong et al., 2019). In addition to the ECM of IPF patients, TGF-β, endothelin, CTGF, interleukins (IL-3 and IL-4), serum response factors (Shi-Wen et al., 2004) and microRNA (miRNA-21, miRNA-22, miRNA-29, miRNA-125b, miRNA-126, miRNA-130a and miRNA-132, miRNA-142a) (Pandit et al., 2011; Wang et al., 2012; Cushing et al., 2015; Kuse et al., 2020) have been shown to be associated with the differentiation of fibrocytes into fibroblasts. After getting activated, fibroblasts proliferate, differentiate, resist apoptosis and can directly lead to IPF through the secretion of profibrotic factors and the remodeling of the ECM (Selman et al., 2000). Under normal circumstances, the secretion and degradation of the ECM by fibroblasts are in a dynamic balance. However, when exposed to inflammation and environmental stress, fibroblasts are reprogrammed and continue to be active and resist apoptosis (Filer and Buckley, 2013), ultimately leading to an increase in the relative rate of ECM synthesis. In patients, the number of fibrocytes and fibroblasts in the lung is positively correlated with collagen deposition and the progression of pulmonary fibrosis. When fibroblast foci start to cross-link with each other, patient lung function may decrease substantially (Snijder et al., 2019).

Although fibroblast is the chief culprit in pulmonary fibrosis, its source is still unclear. Possible sources include peripheral recruitment, mesenchymal progenitor cells in lung tissues, and EMT (Figure 2C). When local lung injury occurs, epithelial cells and endothelial cells are activated and release chemokines. Through chemokine ligand–receptor pathways (including CXCL12/SDF1-CXCR4, CCL21-CCR7 and CCL2-CCR2) (Chong et al., 2019), many circulating fibrocytes and mesenchymal progenitor cells are recruited into local tissues and undergo phenotypic transformation to become fibroblasts. In the lungs of IPF patients, there is a high level of CXCL12/CXCR4 (Mehrad et al., 2007), which is conducive to the recruitment of circulating fibrocytes to the lungs along the concentration gradient of chemokines. In addition, AECs that cannot re-epithelialize can convert into interstitial AECs through EMT, leading to a decrease in intercellular junctions and epithelial features. However, the contribution of epithelial EMT in pulmonary fibrosis is still controversial (Salton et al., 2020). In addition, local fibrocytes and fibroblasts can self-proliferate rapidly. Under the influence of cytokines, growth factors and TIMPs, local fibroblasts in IPF can resist apoptosis and continue to proliferate (Andersson-Sjöland et al., 2011).

### 3.4 Extracellular Matrix

In IPF, ECM remodeling and collagen deposition are the classical pathological features of the disease. ECM is mainly produced by fibroblasts/myofibroblasts, epithelial cells, inflammatory cells, and mesenchymal progenitor cells.

**FIGURE 3:**
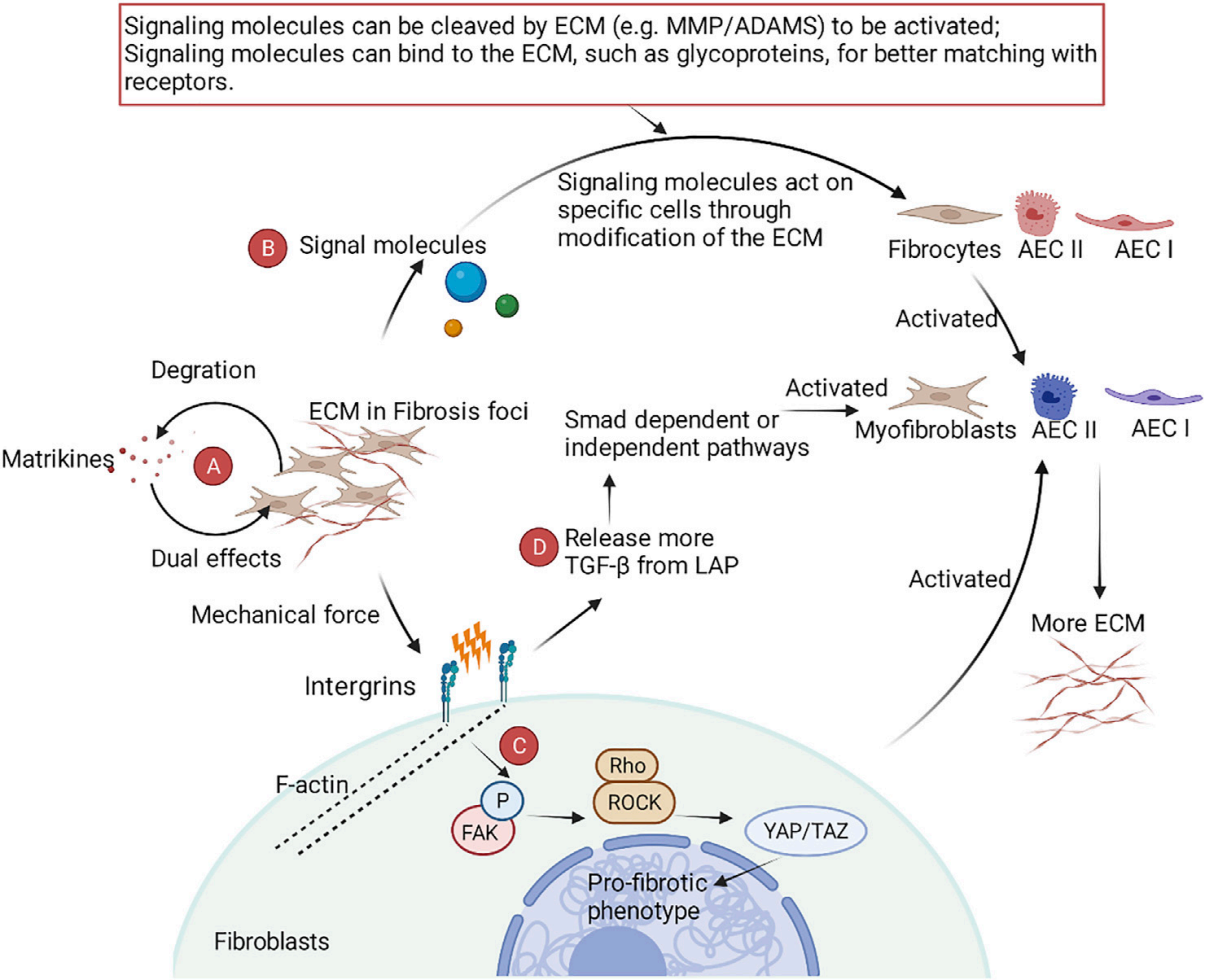
A schematic view of the role of ECM in IPF. **(A)** Matrkines, which contain multiple degradation products of the ECM, exert dual pro- and anti-fibrotic roles in IPF. **(B)** The ECM is involved in the phenotypic reprogramming of fibroblasts and AECs by modifying key signaling molecules. **(C)** After binding specific ECM ligands to the ectodomain of integrins, integrins bind to the cytoskeleton and various signaling proteins through their cytoplasmic tails, translating the mechanical force of cytoskeleton contraction and ECM stiffness into biochemical signals. **(D)** Integrins activate TGF-β-dependent fibrosis by releasing TGF-β through stretch force generated by actin-cytoskeleton interactions.

ECM has complex functions in IPF (Figure 3). ECM has a certain degree of mechanical stiffness, which plays an important role in the sclerosis of lung tissues (Elowsson Rendin et al., 2019). Besides, ECM also serves as a pool for variety of growth factors (bFGF, VEGF), stimulating factors (GM-CSF, M-CSF), and interleukins (IL-1, IL-8), allowing signal exchange with different cells (Andersson-Sjöland et al., 2011). The mechanical force of the ECM itself can also directly participate in pulmonary fibrosis through mechanoreceptors and some cellular pathways. Under the action of mechanical force, α6 integrin (Rahaman et al., 2014) and the transient receptor potential vanilloid 4 (TRPV4) channel (Chen et al., 2016) act as sensors to detect mechanical stimulation signals and transfer the signals into myofibroblasts. Subsequently, F-actin contracts, resulting in the phosphorylation of focal adhesion kinase (FAK). Phosphorylated FAK activates Rho kinase (ROCK) by binding Rho to ROCK. This step activates yes-associated protein (YAP)/transcriptional coactivator with PDZ-binding motif (TAZ), which ultimately activates the transcription, translation, and expression of profibrotic genes in the nucleus. In addition to the FAK-ROCK-YAP/TAZ axis, mechanical force can also release TGF-β bound by latency-associated peptide (LAP), thus activating the TGF-β-Smad dependent/independent pathway in fibroblasts and AECs and directly participating in myofibroblast differentiation (Upagupta et al., 2018).

Matrikines, the degradation product of the ECM, also have special biological activities in lung diseases (Burgess and Weckmann, 2012). Matrikines are biologically active fragments derived from the degradation of ECM. These biologically active fragments exert dual biological properties by binding to integrins, heparan sulfate proteoglycans, and growth factor receptors. For example, endotrophin (the degradation product of collagen VI) promotes fibrosis, while endostatin (the degradation product of collagen XVIII) has antifibrosis effects (Ricard-Blum and Salza, 2014).

ECM includes the core matrisome and associated matrisomes. Core matrisomes include collages, ECM glycoproteins, and ECM proteoglycans. Associated matrisomes include ECM regulators, ECM-affiliated proteins, and secreted factors (Kreus et al., 2021). In the ECM of IPF patients, laminins and collagen IV associated with the basement membrane decreased, while the vast majority of core matrisomes showed an increasing trend (Elowsson Rendin et al., 2019). Although there have been numerous studies, very little is known about the role of some mediators in IPF. The various substances in the ECM are described in detail below.

#### 3.4.1 Collagen

Collagen is the main component of ECM. Collagen is derived from activated (myo)fibroblasts, fibrocytes, epithelial cells (which can transform into mesenchymal cells through EMT), mesenchymal stromal cells, and pericytes. The vast majority of interstitial and fibrillar collagens (mainly collagen I and collagen III) are secreted by (myo)fibroblasts. These collagens constitute the main frame of the ECM and increase the mechanical stiffness of fibrotic tissue (Snijder et al., 2019). Currently, it is believed that type III collagen plays a role in the early disease stage and that type I collagen is associated with the deterioration of lung function in the late disease stage due to its involvement in collagen crosslinking mediated by lysyl oxidase (LOX) family enzymes (Rock et al., 2011). In view of the important contribution of collagen to the mechanical stiffness of pulmonary fibrosis, collagen turnover has naturally become an important means for monitoring disease progression and predicting patient prognosis. Increased concentrations of the collagen degradation markers type 1/3/6 collagen, and C-reactive protein are associated with an increased risk of overall mortality, and elevated levels of the collagen synthesis markers Pro-C3 and Pro-C6 are associated with IPF progression (Organ et al., 2019; Jessen et al., 2021).

#### 3.4.2 Glycoproteins and Proteoglycans

There are relatively few studies on ECM glycoproteins and proteoglycans. In a radiation-induced pulmonary fibrosis model, the glycoprotein glectin-3 produced by type I AECs increased dramatically (Kasper and Hughes, 1996). In recent years, studies on proteoglycans have shown that the proteoglycan decorin reduces pulmonary fibrosis by antagonizing TGF-β and antagonizing CTGF-mediated collagen deposition (Nikaido et al., 2018). In addition, the proteoglycan lumican directly acts in the differentiate of monocytes into fibroblasts through an integrin-dependent pathway (Pilling et al., 2015). Chondroitin sulfate type E (CS-E), another proteoglycan, inhibits the expression of α-SMA, CTGF, LOXL2, and CCL2/MCP-1 by silencing the genes of CS-E and the related enzyme carbohydrate sulfotransferase 15 (CHST15) through miRNA (Kai et al., 2017). Syndecan-4 is a heparan sulfate proteoglycan. Silencing of syndecan-4 can reduce SMA-α and collagen deposition (Tanino et al., 2019). In addition, proteoglycans assist other factors in producing biological effects. For example, FXIIa must bind to the proteoglycan heparan sulfate to stimulate the migration of human lung fibroblasts (Wujak et al., 2015).

#### 3.4.3 ECM Regulators

ECM regulators include serine protease inhibitors, cystatins, TIMPs, MMPs (Kreus et al., 2021), a disintegrin and metalloproteinases (ADAMs), and crosslinking enzymes (Ricard-Blum and Salza, 2014; Liu et al., 2016); these molecules are mainly involved in the regulation of ECM decomposition. MMPs/TIMPs play an important role in IPF and are directly involved in ECM remodeling. MMPs belong to zinc-dependent endopeptidases of the M10A subfamily, and 24 gene subtypes are expressed in the human body. As a crucial component of ECM, MMPs participate in the formation and progression of IPF via many pathways, especially TGF-β signaling pathway. Many studies have focused on MMPs and explored their potential value in resisting IPF (Table 1). Although the pathological processes of MMPs and IPF have been verified, there are no MMP inhibitors for the treatment of IPF in the clinic (Yue et al., 2021).

**TABLE 1:** The potential role of MMPs/TIMPs in IPF.

MMPs	Cellular Sources	Pro/Antifibrotic	Mechanism	Ref
MMP1	AEC, macrophage	Profibrotic	MMP1 Induces lung alveolar epithelial cell migration and proliferation, protects from apoptosis, and represses mitochondrial oxygen consumption by activation of HIF-1α	Herrera et al. (2013)
MMP2	BECs, AECs, fibroblasts, fibrocytes	Profibrotic	MMP2 damages the integrity of alveolar walls, regulates EMT, and involves in activation of TGF-β	Nguyen et al. (2001); Wu et al. (2007); Lam et al. (2014)
MMP3	BECs, AECs, macrophage, fibroblasts	Profibrotic	MMP3 activates the β-catenin and TGF-β pathway, induces EMT, releases endothelin	Wu et al. (2007); Richter et al. (2009); Yamashita et al. (2011)
MMP7	Lung epithelial cell, fibroblast	Pro/Antifibrotic	MMP7 regulates osteopontin, mediates E-cadherin ectodomain shedding, and regulates anti-inflammatory and antifibrotic pulmonary dendritic cells	McGuire et al. (2003); Pardo et al. (2005); Manicone et al. (2009)
MMP8	blood monocytes, AM, BECs, AECs, fibrocytes	Profibrotic	MMP8 reduces the levels of IL-10, IP-10 and MIP-1a, affects the migration of fibrocytes mediated by PDGF-B or stromal cell-derived factor-1α	García-Prieto et al. (2010); Craig et al. (2013)
MMP9	AECs, neutrophils, AM, fibrocytes, fibroblasts	Profibrotic	MMP9 production is related to TGF-β1. MMP9 cleaves SP-D; MMP9 involves in macrophage-induced fibroblast migration and TGF-β1/Smad2-dependent EMT.	Murthy et al. (2010); Bratcher et al. (2012); Li et al. (2019); Zhang et al. (2019)
MMP10	AM, BECs, AECs	Antifibrotic	MMP10 involves in the migration of macrophages and macrophage-mediated collagenase degradation	Murray et al. (2013); Rohani et al. (2015)
MMP11	furin-like proconvertase enzyme	Profibrotic	MMP11 activates Notch pathway and promotes fibroblasts to myofibroblasts differentiation	Aoyagi-Ikeda et al. (2011)
MMP14	AECs, AM, and endothelial cells	Antifibrotic	MMP14 involves in activation of TGF-β, fibroblast-dependent collagenolysis and invasion	Sabeh et al. (2009); Xiong et al. (2017)
MMP19	monocytes, AM, fibrocyte, AEC	Antifibrotic	MMP19 promotes wound healing and cell migration by inducing PTSG2 (prostaglandin endoperoxide synthase 2); MMP19 regulates ECM formation, migration, proliferation, and autophagy of fibroblasts	Yu et al. (2012); Jara et al. (2015)
MMP28	Macrophages	Profibrotic	MMP28 promotes M2 polarization and TGF-β-dependent EMT.	Jara et al. (2015)

ECM contains a variety of signal molecules secreted by cells; therefore, the ECM is a transfer station for signal exchange. In IPF, the signaling molecules from different cells constitute a complex signaling network through the bridge function of the ECM. Upstream signals are modified and regulated by positive feedback in the ECM; this feedback, e.g., the TGF-β pathway and Rho/ROCK signaling, can affect the transcription and translation of ECM-related genes and eventually lead to pulmonary fibrosis (Snijder et al., 2019).

Although there have been many studies on active fragments, the roles of these active products in IPF are still not clear. In recent years, the ECM has shown extraordinary clinical and scientific value in the fields of anti-infection, anti-angiogenesis, and wound healing (Ricard-Blum and Salza, 2014; Ricard-Blum and Vallet, 2019). Therefore, research on active fragments and their derivatives as targets for antifibrosis therapies is innovative and has great potential.

## 4 Underlying Factors Associated With the Pathogenesis of IPF

### 4.1 Genetic Susceptibility and Epigenetic Alterations in the Development of IPF

A growing number of studies have shown that genetic mutations are associated with susceptibility, diagnosis, progression, prognosis, and adverse effects of treatment in IPF. The proportion of patients carrying genetic mutations in IPF may be underestimated. A cohort study showed that up to 36% of patients with IPF have familial genetic mutations (Barros et al., 2019). In addition, IPF mutations are present in nearly 40% of sporadic pulmonary fibrosis cases (Wolters et al., 2014). These common and rare genetic mutations mainly include mutations in telomeres, alveolar surfactant and muco-ciliary transportation system, immune and cytokine-related genes, cell adhesion and cell integrity-related genes (Table 2). MUC5B and telomere related gene mutations are more common in IPF patients, which has inspired a very large number of studies to explore the pathogenic mechanisms of gene mutations (Evans et al., 2016; Zhang et al., 2021). Besides, genetic variants have been associated with the imaging presentation of IPF. Patients with TERT mutations were more likely to exhibit the classical UIP pattern compared to patients without mutations with two-year-follow up (Baratella et al., 2021).

**TABLE 2:** Genetic mutations associated with IPF.

Targets	Physiological function of the target site	Potential pathogenic mechanisms of gene mutations	Clinical significance	Ref
TERT and TERC	TERT and TERC are important components of the telomerase complex	Telomere shortening may affect the turnover and healing of AEC.	TERT (rs2736100) and TERC (6793295) mutations are associated with IPF susceptibility	Armanios et al. (2007); Borie et al. (2016)
DKC1	DKC1, a pseudouridine synthase, is involved in the synthesis of non-coding ribonucleic acids	Mutations in DKC1 can shorten telomeres in alveolar epithelial cells and affect the stability of telomerase RNA.	DKC1 mutations cause dyskeratosis congenita and pulmonary fibrosis	Kropski et al. (2017); Gaysinskaya et al. (2020)
TIN2	TIN2 is an important component of the shelterin complex	Mutations in TIN2 can shorten telomeres	Heterozygous mutations in TINF2 causes IPF	Fukuhara et al. (2013)
PARN	PARN, a 3′exoribonuclease, is responsible for telomere maturation	PARN mutations lead to shortened telomeres	PARN mutations and telomere shortening are associated with leukopenia	Stuart et al. (2015)
RTEL	RTEL is a DNA helicase crucial for unwinding the T-loop structure	Loss of functional RTEL1 leads to cleavage of the telomeric end proximal to the T-loop by endonuclease SLX4, leading to release of T-loops and shortened telomere	RTEL and telomere shortening are associated with leukopenia	Cogan et al. (2015); Stuart et al. (2015)
NAF1	NAF1, a box H/ACA RNA biogenesis factor, is required for stability and assembly into a mature telomerase holoenzyme complex	NAF1 mutations can reduce telomerase RNA levels, resulting in shorter telomeres	Pulmonary fibrosis-emphysema in NAF1 mutation patients is telomere-mediated	Stanley et al. (2016a)
OBFC1	OBFC1 associates with TPP1 and is implicated in telomere length regulation	N.A.	rs11191865 was associated with a lower risk of IPF.	Fingerlin et al. (2013)
MUC5B	Mucin 5B is involved in mucosal clearance along with surfactant protein C and ciliates	Excess Mucin may increase the retention of harmful particles in the lung and interfere with the normal developmental pathway and alveolar epithelial repair	rs35705950 was the strongest genetic risk factor for IPF, but was associated with lower mortality. MUC5B and MUC5AC expression was increased in patients with IPF.	Peljto et al. (2013); Conti et al. (2016); Evans et al. (2016)
SFTPC	SFTPC regulates alveolar surface tension	SFTPC mutations may promote lung fibrosis by inducing endoplasmic reticulum stress and apoptotic cell death in AEC II.	SFPTC mutations are associated with familial and sporadic IPF onsets	Ono et al. (2011); Venosa et al. (2017)
SFTPA2	SFTPA is involved in the intrinsic immunity of the lung	SFTPA mutant mouse models exhibit intracellular retention of SFTPA and enhanced ER stress	Mutations in SFTPA2 leads to the trafficking of several proteins and causes the development of IPF.	Yongyu Wang et al. (2009); Maitra et al. (2010); Guenther et al. (2019)
ABCA3	A type of phospholipid carrier, involved in the secretion and transport of surface-active substances in AEC II.	ABCA3 mutations may induce ER stress and proteostasis failure through misfolded alveolar surface-active substances	Heterozygous variants of the ABCA3 gene are associated with IPF susceptibility. pG1205R, an ABCA3 gene allele, is more frequently expressed in patients with IPF and ILDs	Zhou et al. (2017); Manali et al. (2019)
ATP11A	ATP11A encodes ABCA1, a transmembrane protein with general transport function	N.A.	rs1278769 was associated with a lower risk of IPF.	Fingerlin et al. (2013)
IL1RN	IL-1RN is a competitive antagonist of IL-1R receptor	MSC exerts anti-inflammatory and anti-fibrotic effects via IL-1RN.	The proportion of IL-1RN gene polymorphisms in patients with fibrosing alveolitis was more	Whyte et al. (2000); Ortiz et al. (2007)
IL-4	IL-4 is associated with type 2 immunity	IL-4 gene polymorphisms may promote a Th2 cytokine environment with exaggerated fibroproliferative healing	Higher percentage of IL-4 gene polymorphisms in IPF patients	Vasakova et al. (2013)
IL-8	IL-8 is a chemokine secreted by macrophages and is involved in the recruitment of neutrophils	IL-8 increases the fibrogenicity of mesenchymal progenitor cells and is involved in the proliferation, activation, and recruitment of mesenchymal cells	IL-8 gene diversity is associated with lung alveolitis and lung function decline	Ziegenhagen et al. (1998); Yang et al. (2018)
TLR3	TLR3 is known as one of the innate immunity receptors, which mediate inflammation, tissue injury and viral infection	Defective TLR3 L412F gene activates abnormal inflammation and promotes fibroplasia in IPF, which may be associated with dysregulation of fibroblast proliferation mediated by a sluggish IFN-β response	rs3775291 increase the risk for IPF patients and also reduces forced volume capacity (FVC)	O'Dwyer et al. (2013); O'Dwyer et al. (2015); Evans et al. (2016)
TOOLIP	TOLLIP is involved in the signaling pathway of TGF-β, TLR and ILs	rs3750920 may lead to unregulated TLR signaling pathway	rs5743890 was associated with a lower susceptibility to IPF, whereas rs5743894 was associated with a higher susceptibility to IPF. The rs3750920 polymorphism was associated with the efficacy of NAC. rs5743890 was associated with increased IPF morbidity and mortality	Noth et al. (2013); Oldham et al. (2015)
HLA-DRB1	HLA gene encodes major histocompatibility complex (MHC)	N.A.	HLA-DRB1*1501 is related to greater differences in gas exchanges and immunogenic process	Xue et al. (2011); Zhang et al. (2015)
MDGA2	MDGA2 encodes a paralogue for ICAM, which has been shown to be a potential biomarker of IPF disease activity	N.A.	rs7144383 was associated with a higher risk of IPF.	Noth et al. (2013)
DSP	DSP, a desmosomal protein, is mainly expressed in the airway epithelium and is involved in cell adhesion	rs2076304 might influence the binding of RHOXF1	rs2076304 and rs2076295 increased the IPF risk and rs2744371 decreased the IPF sub-risk	Mathai et al. (2016); Wang et al. (2018)
DPP9	DPP9 is a serine protease that belongs to a member of the S9B family. DPP9 is expressed in epithelial cells and is involved in cell adhesion, cell migration and apoptosis	N.A.	rs12610495 is associated with IPF susceptibility	Fingerlin et al. (2013); Zhou and Wang, (2016)
SPPL2C	SPPL2C is a transmembrane GxGD type of cleavage proteases	N.A.	rs17690703 was also known to reduce FVC in IPF. A low survival rate and mortality were reported in people with greater gene SPPL2C expression	Wu et al. (2016); Lorenzo-Salazar et al. (2019)
AKAP13	AKAP13 is a Rho guanine nucleotide exchange factor regulating activation of RhoA	AKAP13 mutations may affect the RhoA/ROCK signaling pathway	rs62025270 was associated with increased production of AKAP13, but no correlation with survival was observed	Allen et al. (2017)
FAM13A	FAM13A contains a protein domain called Rho GTPase activating protein (Rho GAP)	FAM13A mutation may affect the RhoA/ROCK signaling pathway	The rs2609255 was associated with higher mortality rate. The FAM13A allele was associated with worse disease and lower DLCO.	Hirano et al. (2017); van Moorsel, (2018); Ruffin et al. (2020)
MAPT	MAPT encodes Tau protein, a microtubule-associated protein	N.A.	rs1981997 is associated with a lower risk of IPF.	Fingerlin et al. (2013); van Moorsel, (2018)

Notably, the majority of existing studies still remain to verify the correlation between mutations and IPF susceptibility, and there are only a few studies related to the causality and pathogenesis of IPF. Moreover, further research needs to focus on how to cross the gap between genetic testing and clinical treatment. Drugs targeting histone modifications and DNA modifications have also shown potential in preclinical studies, so we will also cover the role of epigenetics in IPF in the next sections.

#### 4.1.1 Genetic Mutations in Telomeres

Telomeres are a TTAGGG repeat sequence at the end of human chromosomes, whose function is to stabilize the chromosome, prevent fusion of chromosome ends, protect structure of chromosomes, and determine cell life span, but telomerase activity in adult human cells is extremely low (Lister-Shimauchi et al., 2022).

Telomeres are protected by the shelterin complex, which consists of the protein components TRF1, TRF2, RAP1, TIN2, TPP1, and POT1. The ends of telomeres are bonded with telomerase, which is comprised of the catalytic subunit of TERT, the telomerase RNA component of TERC, and accessory proteins. The telomerase exerts its effects by adding telomeric sequences to the telomeric ends (Zhang et al., 2021). Telomere shortening is one of the important phenotypes of IPF. Telomere length of half of IPF patients rank in the lowest 1% of their age group (Stanley et al., 2016b). And telomeres are shorter in IPF patients than in other interstitial pneumonia (Snetselaar et al., 2015). However, it is questionable whether the cause of telomere shortening in IPF patients necessarily stems from telomere gene mutations. In patients with sporadic and familial pulmonary fibrosis, telomere shortening is present regardless of the presence of known telomere related gene mutations (Snetselaar et al., 2015). A possible reason for this is the presence of other unknown genetic mutations that can shorten telomere length.

The causal relationship between mutation-mediated telomere shortening and the development of IPF is inconclusive because not all individuals carrying a known telomere defective gene develop telomere shortening and IPF (Hoffman et al., 2018). Firstly, telomere gene defects in different cells may lead to different outcomes. systematically TERT knock-out does not induce spontaneous pulmonary fibrosis in mice, but can increase susceptibility to bleomycin (Degryse et al., 2012), while mesenchymal-specific TERT knock-out can alleviate bleomycin-induced pulmonary fibrosis (Liu et al., 2015); and AEC II-specific TERT defect reduces AEC II proliferation and induces AEC II cell senescence (Liu T. et al., 2019). Secondly, telomere gene defect-mediated DNA damage may be slow, which requires multiple generations of mice to become apparent (Blasco et al., 1997). Thirdly, due to the heterogeneity of the clinical manifestations, even the same genetic mutations may have different phenotypes, leading to the appearance of extra-pulmonary pathological features (rather than interstitial lung disease) (Borie et al., 2016). Fourthly, telomere length may differ in peripheral blood leukocytes and lung tissue (Snetselaar et al., 2017). Therefore, before telomere length measurements can be applied to clinical decisions, there is a need to clarify the effect of telomere length on different cells, the optimal sample type, and the corresponding intrapulmonary/extrapulmonary clinical manifestations.

The mechanism by which telomerase gene mutations lead to IPF is unclear. Available data suggest that cellular senescence or death of alveolar stem cells induced by telomere dysfunction is implicated in pulmonary fibrosis (Zhang et al., 2021). Knockdown of telomerase related genes leads to lung stem cell regenerative capacity and the senescence/death of AEC II (Jackson et al., 2011; Alder et al., 2015; Snetselaar et al., 2017; Wang et al., 2020). In addition, knockdown of telomerase related genes leads to elevated SASP (including TGF-β) levels and lung inflammation mediated by intrinsic immune cell infiltration (Chen et al., 2015; Povedano et al., 2015; Naikawadi et al., 2016; Liu et al., 2018).

Telomere length and specific telomere gene defects are instructive for susceptibility, diagnosis, progression, prognosis, and early warning of adverse effects in IPF. Shorter telomere length as an independent predictor is associated with worse survival and more pronounced imaging changes in IPF patients (Stuart et al., 2014; Newton et al., 2016; Ley et al., 2017). Pirfenidone treatment did not improve FVC and DLCO in IPF patients carrying TERT/TERC mutations (Justet et al., 2018). The relationship between TERT/TERC mutations and resistance to drug therapy needs to be further validated. Patients carrying telomere-related mutations may be at higher risk for complications after lung transplantation, such as death, chronic lung allograft dysfunction, renal disease, CMV complications (Popescu et al., 2019; Swaminathan et al., 2019), hematological complications (Silhan et al., 2014; Borie et al., 2015), anemia, leukopenia, recurrent lower respiratory tract infections (Tokman et al., 2015).

Drugs that target telomeres have also shown therapeutic potential. GRN510, a telomerase agonist, reduces inflammatory infiltration and collagen deposition in a mouse model of bleomycin-induced pulmonary fibrosis (Le Saux et al., 2013). PAP-associated domain-containing protein 5 (PAPD5) is involved in the degradation of TERC RNA. BCH001 (a PAPD5 inhibitor) and RG7834 (a PAPD5/7 inhibitor) rescue the TERC levels and telomerase activity in the cellular model of dyskeratosis congenital (Nagpal et al., 2020; Shukla et al., 2020). One study reported that hormones may regulate telomerase activity through an imperfect oestrogen response element within the TERT promoter (Bayne and Liu, 2005), which has inspired a series of subsequent clinical trials of hormones for IPF. Danazol, a synthetic androgen, was shown to increase telomere length and stabilize DLCO and FVC in a small-scale clinical trial (Townsley et al., 2016a; Townsley et al., 2016b). Danazol is currently in phase II clinical trials for plumonary fibrosis (NCT04638517). However, hepatotoxicity and worsening pulmonary fibrosis associated with long-term use of Danazol has been reported after danazol initiation and withdrawal (Chambers et al., 2020). Therefore, lower doses of danazol (200 mg) are being used in clinical trials to reduce hepatotoxicity. Nandrolone Decanoate, an anabolic androgenic steroid, is undergoing phase I/II clinical trials (NCT02055456). Telomerase reactivation therapy still has a long way to go before it becomes a therapeutic option, especially in terms of the serious adverse effects associated with hepatotoxicity. Whether peripheral blood telomere length can be used as a substitute outcome for pulmonary telomere length and how effective is telomerase reactivation therapy for IPF are still awaiting the results of current clinical trials.

In summary, details remain unclear in the complex field of the role telomeres play in the pathogenesis of ILDs and no clinical trials employing gene therapy have been initiated in patients with telomeropathies. Hopefully, adeno-associated virus-9 (AAV9)-mediated gene therapy has been reported to be successful in mouse models (Bär et al., 2016), and perhaps a telomerase-directed therapeutic strategy may be used for the treatment of IPF in the future.

#### 4.1.2 Genetic Mutations in Alveolar Surfactant/Mucin

**FIGURE 4:**
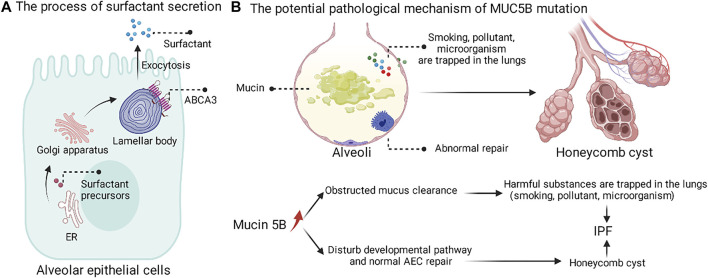
The process of surfactant secretion and the potential pathological mechanism of MUC5B mutation. **(A)** The surfactant precursors are synthesized on the endoplasmic reticulum, followed by Golgi modifications, and finally secreted into the periphery by cytosolic exocytosis of the lamellar body. **(B)** MUC5B mutation may promote the retention of harmful substances in the lung and interfere with the normal repair of AEC.

Phospholipids and four surfactant proteins (SFTPA, B, C, D) can be secreted by AEC II. The process of surfactant secretion is shown (Figure 4A). ATP-binding cassette subfamily A member 3 (ABCA3) is essential for the intracellular synthesis of alveolar surface-active substances (Mulugeta et al., 2015). Among four surfactant, genetic mutations of SFTPA (Wang Y. et al., 2009; Guenther et al., 2019), SFTPC (Venosa et al., 2017) and ABCA3(Zhou et al., 2017; Manali et al., 2019) have been identified in IPF patients. Although SFTPA is not directly involved in the formation of alveolar surface-active tension, it plays an important role in intrinsic lung immunity. Mouse models with SFTPA mutations exhibit intracellular retention of SFTPA and enhanced ER stress (Wang Y. et al., 2009; Maitra et al., 2010). Molecular signatures of UPR signaling and apoptotic activation associated with SFTPC have been reported in patients with IPF (Korfei et al., 2008; Lawson et al., 2008). Patients with pure mutations in ABCA3 and ABCA3-deficient mice exhibit a complete lack of alveolar surface-active substance, deformation of lamellar bodies, and death from respiratory distress shortly after birth (Shulenin et al., 2004; Cheong et al., 2007; Fitzgerald et al., 2007; Wambach et al., 2014). In lung disease, there are also reports of ABCA3 heterozygous mutations that appear to interact with SFTPC mutations (Bullard and Nogee, 2007; Crossno et al., 2010). ABCA3 mutations may induce ER stress and proteostasis failure through misfolded alveolar surface-active substances (Cheong et al., 2006; Matsumura et al., 2006; Weichert et al., 2011).

The MUC5B promoter variant, rs35705950, as the strongest genetic risk factor and common variation, was found in up to 30% of IPF (Evans et al., 2016). The rs35705950 mutation leads to increased Muc5B expression, but a causal relationship between mucus and progression of pulmonary fibrosis disease has not been established. Christopher M. Evans proposed two hypotheses for the pathogenic mechanism of MUC5B mutations (Figure 4B) (Evans et al., 2016). The first possible mechanism is that overexpression of Muc5B at the bronchoalveolar junction impairs mucociliary clearance, which in turn promotes prolonged retention of harmful particles (e.g., air pollutants, cigarette smoke, microorganisms, etc.) in the lung and induces fibrosis in the lung tissue. The second possible mechanism is that overexpression of Muc5B may lead to fibrosis and cellulitic cyst formation by interfering with the normal developmental pathway and alveolar epithelial repair. In summary, the overexpression of MUC5B at the bronchoalveolar junction in IPF patients led us to recognize that the peripheral airway also seems to influence interstitial fibrosis.

#### 4.1.3 Genetic Mutations in Inflammatory Regulators

Genetic mutations in cytokines have also been reported in patients with IPF. IL1RN gene polymorphisms may be associated with susceptibility to fibrosing alveolitis, a disease characterized by interstitial lung fibrosis (Whyte et al., 2000). Mesenchymal stem cells (MSC) can exert anti-inflammatory and anti-fibrotic effects via IL-1RN (Ortiz et al., 2007). Association between genetic polymorphisms of IL-4 and IPF has been reported (Riha et al., 2004). IL-4 may be pro-fibrotic by promoting type 2 immunity. IL-8 gene diversity is associated with lung alveolitis and lung function decline (Ziegenhagen et al., 1998). IL-8 increases the fibrogenicity of mesenchymal progenitor cells and is involved in the proliferation, activation, and recruitment of mesenchymal cells (Yang et al., 2018). TLR3 (Toll-like receptor 3) mutation rs3775291 increase the risk for IPF patients and also reduces forced volume capacity (FVC) (Evans et al., 2016). Defectiveness of the gene TLR3 L412F causes aberrant inflammation and fibroblasts proliferation in IPF, which may be related to dysregulation of fibroblast proliferation mediated by a sluggish IFN-β response (O'Dwyer et al., 2013). The different mutations of TOOLIP (TOLL interacting protein) may be associated with different IPF susceptibility. The rs5743890 was associated with lower IPF susceptibility, while the rs5743894 was associated with higher IPF susceptibility (Oldham et al., 2015). In addition, rs5743890 was associated with increased morbidity and mortality in IPF (Noth et al., 2013). rs3750920 polymorphism is associated with the efficacy of N-Acetyl-L-cysteine (NAC) (Oldham et al., 2015). It is important to note that different studies with different populations and specimens may reach different conclusions. In a study conducted in China, except for the polymorphisms of HLA haplotype, none of the other cytokines showed an association for IPF susceptibility (Zhang et al., 2015). Therefore, the study of genetic polymorphisms of cytokines should pay more attention to the reproducibility of experimental results.

In recent years, the launch of pirfenidone and nintedanib has witnessed breakthroughs in the signaling pathways of cytokines and growth factors in the treatment of IPF, however, studies focusing on cytokine and immune-related gene polymorphisms need to be further developed.

#### 4.1.4 Other Potential Genetic Mutations Associated With IPF

In recent years, with the availability of next-generation sequencing technologies, more and more novel mutations have been identified in IPF patients. The mutations of the gene DSP (Mathai et al., 2016; Wang et al., 2018) and DPP9 (associated with cell adhesion) (Fingerlin et al., 2013; Zhou and Wang, 2016), AKAP13 166] and FAM13A (associated with RhoA/ROCK signaling pathway) (Hirano et al., 2017; van Moorsel, 2018), and SPPL2C (associated with intramembrane proteases) (Wu et al., 2016; Lorenzo-Salazar et al., 2019) were reported to be a risk of IPF susceptivity, progression and prognosis. However, the pathogenesis of these mutations remains unclear, and current studies are limited to correlation analysis (Table 2).

#### 4.1.5 DNA Methylation

DNA methylation is a chemical modification process in which a specific base on the DNA sequence acquires a methyl group by covalent bonding with S-adenosyl methionine (SAM) as the methyl donor. DNA methylation is catalyzed by DNA methyltransferase (DNMT). In humans, DNA methylation occurs mainly at the fifth carbon atom of cytosine in CpG dinucleotides. It is generally believed that DNA methylation can inhibit the transcription and expression of the gene.

**FIGURE 5:**
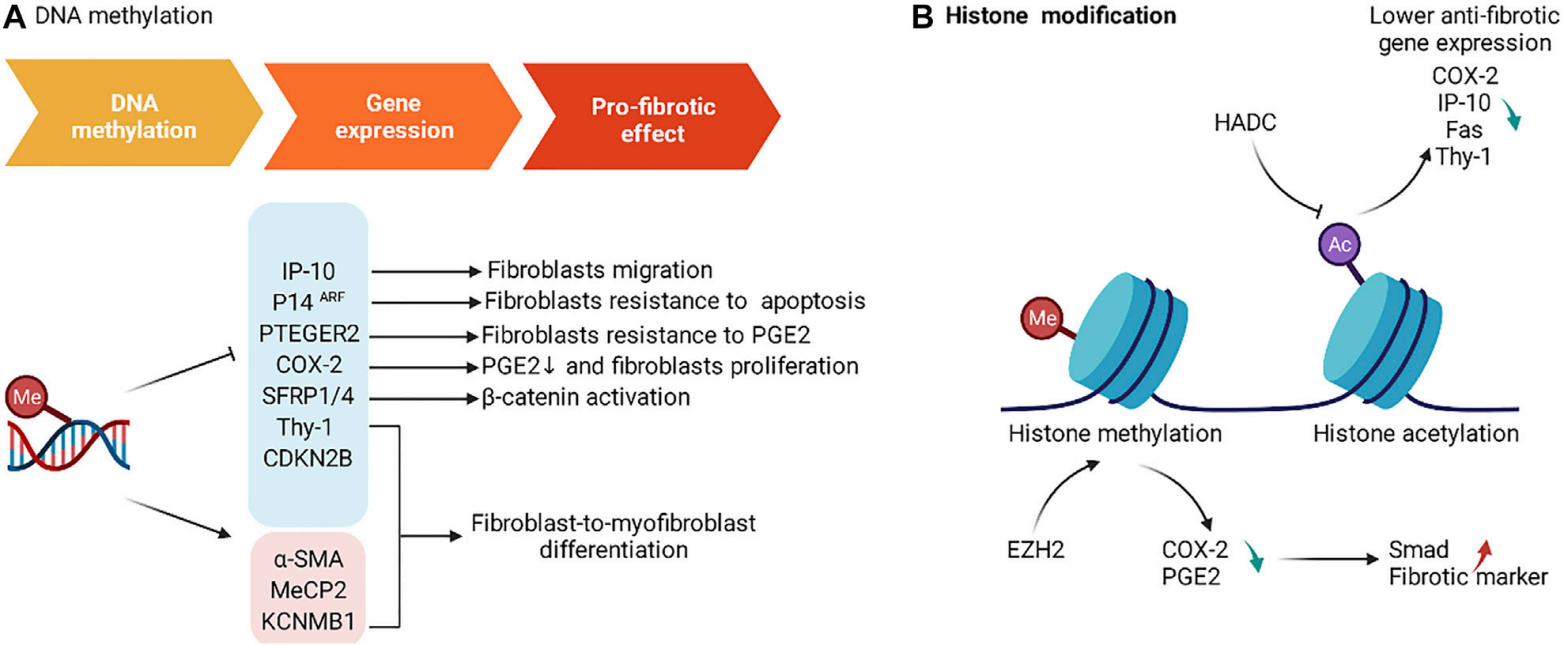
The roles of DNA methylation and histone modification in IPF. **(A)** DNA methylation affects the expression of gene associated with fibrosis and produces a pro-fibrotic effect. **(B)** EZH2 and HADC induce changes in gene expression associated with fibrosis by affecting histone modifications.

Global methylation and methylation of specific gene have been shown to be involved in the pathogenesis of IPF. Global DNA methylation analysis based on whole lung tissue showed that DNMT expression was increased in IPF, and DNA methylation of 16 genes (including CLDN5, ZNF467, TP53INP1 and DDAH1) was associated with decreased mRNA expression (Sanders et al., 2012). In order to clarify the contribution of specific cell types to IPF, single-cell-based or cell-type-specific sequencing is necessary. The global DNA methylation analysis based on fibroblasts showed that cells derived from IPF patients differ in the methylation of multiple CpG sites (including CDKN2B, CARD10, and MGMT) (Huang et al., 2014). Hypermethylation of specific genes not only can suppress the expression of some genes [Thy-1, COX-2, PTGER2, p14^ARF^, IP-10 (Zhou et al., 2020), SFRP1/4, CDKN2B (Xue et al., 2021)], but also can promote the expression of some genes [α-SMA, MeCP2, KCNMB1 (Xue et al., 2021)], which eventually leads to the development of IPF (Figure 5A). Decitabine, a DNMT inhibitor, reduced fibrotic gene and DNMT-1 expression, enhanced miR-17–92 cluster expression (Dakhlallah et al., 2013). PGE2 has the capacity to limit fibrosis through its inhibition of numerous functions of these fibroblasts, and Decitabine restored PGE2 responsiveness in fibrotic fibroblasts (Huang et al., 2010).

#### 4.1.6 Histone Modification

Nucleosome, the basic unit of chromatin, is comprised of 146–147 base pairs of DNA and a histone octamer with one H2A-H2B tetramer and two H3-H4 dimers (Jenuwein and Allis, 2001).

The N- and C-terminal tails of H3 and H4 are the most frequently modified regions. The types of modifications include methylation, acetylation, phosphorylation, SUMOylation, and ubiquitination. The histone mark is named according to the histone, the modification site of these tails, and the type of modification. Histone modifications can change the electronic charge and structure of DNA-bound histone tails, thus altering chromatin state and gene expression (Kouzarides, 2007). The effect of histone acetylation and methylation in IPF has been extensively studied, and a large number of agents have been designed and validated in preclinical studies (Figure 5B).

Histone methylation is catalyzed by the histone methyltransferase (HMT), which uses S-adenosyl methionine (SAM) as the substrate to transfer methyl groups onto the lysine residues of histones. Methylation of H3K4, H3K36, and H3K79 is thought to be associated with gene activation, while H3K9, H3K27, and H4K20 are thought to be associated with gene inactivation (Black et al., 2012). Inhibition of EZH2, an H3K27me-specific methyltransferase, by 3-DZNeP and BIX01294 increased the expression of COX-2 and PGE2, and suppressed the expression of Smad signaling and fibrosis markers (Coward et al., 2014; Xiao et al., 2016).

Histone acetyltransferases (HATs) promote histone acetylation, while histone deacetylases (HDACs) reduce histone acetylation. Histone acetylation is thought to be associated with gene activation (Clayton et al., 1993). In addition, sirtuins (SIRTs) are a family of deacetylases. SIRT1 inhibits the expression of aging-related secretions by deacetylating the SASP gene (Hayakawa et al., 2015). Inhibition of SIRT3 expression promotes fibrosis in human lung fibroblasts (Sosulski et al., 2017). Histone deacetylation is associated with the repression of anti-fibrotic genes, which includes COX-2 (Coward et al., 2009), IP-10 (Coward et al., 2010), Fas (Huang et al., 2013), Thy-1 (Sanders et al., 2011).

Inhibition of histone deacetylation may have anti-pulmonary fibrosis effects. HDACi consists of a zinc-binding-group, a hydrophobic group for protein recognition and interaction, and a linker connecting both of them (Wang and Dymock, 2009). A large number of agents targeting histone modification have already demonstrated antifibrotic effects in preclinical trials or in cell levels (Table 3).

**TABLE 3:** Drugs targeting histone modification in IPF.

Drugs	Targets	Cell or animal model	Mechanism of action	Ref
3-DZNeP	EZH2/G9a inhibitor	BLM mice and LL29 cell	Reduce p-Smad2/3 nuclear translocations *in vitro* and downregulate α-SMA, COL1A1 and COL3A1 expression *in vivo*. Restore COX-2 expression and PGE2 production	Coward et al. (2014); Xiao et al. (2016)
A6	p300i	BLM mice; lung fibroblast cells	Decrease histone acetylation and pro-fibrotic gene expression *in vitro*, and reduce collagen deposition *in vivo*	Hwang et al. (2020)
Ac-SDKP	α-TAT1	MRC5/A549 cell	Promote apoptosis	Shifeng et al. (2019)
Romidepsin	HDACi	BLM mice	Inhibit LOX expression	Conforti et al. (2017)
LBH589	pan-HDACi	Primary IPF	Reduce expression of genes associated with ECM synthesis, proliferation and cell survival and suppress HDAC7 level	Korfei et al. (2015)
CG-745	HDACi	BLM/PHMG mice	Inhibit collagen production, inflammatory cell accumulation, and cytokines release	Kim et al. (2019)
Entinostat and vorinostat	HDACi	HFL-1 cell	Upregulate XPLN mRNA expression and reverse TGF-β-induced SPARC expression	Kamio et al. (2017)
TSA	pan-HDACi	NHLF cell	Reduce p-Akt level to inhibit TGF-β-mediated α-SMA expression	Guo et al. (2009)
SAHA	pan-HDACi	HLF cell	Promote the differentiation of fibroblasts into myofibroblasts and collagen deposition	Z Wang et al. (2009)
SpA	HDACi	Primary IPF	Inhibit the proliferation of IPF fibroblasts by increasing p21 expression	Davies et al. (2012)
JQ1	Bromodomain protein inhibitor	IPF fibroblasts	Bromodomain protein is the “Reader” of acetylated lysine in histone, and it is the only protein domain that can recognize and bind acetylated lysine in histone	Filippakopoulos et al. (2010)

### 4.2 Environment Factors

Environmental factors such as asbestos, metal dust, stone dust, wood dust, chemotherapy and allergen exposure are important causes of IPF (Taskar and Coultas, 2006). Epidemiological surveys have shown that 38% of IPF patients were exposed to high-risk environments; however, it is uncertain whether these factors directly lead to the occurrence of IPF (Behr et al., 2015; Wuyts et al., 2018).

Smoking is a relatively special environmental factor. Both direct smoking and indirect smoking are independent exposure factors, and the correlation between the direct/indirect smoking and IPF increase with the increase of smoking in dose (Bellou et al., 2021). Interestingly, according to the German S2K guidelines, although 60%–70% of patients had a history of smoking, less than 7% of patients were active smokers when they developed IPF symptoms. In other words, most patients developed the disease several years after quitting smoking; the average time interval was 21 years (Behr et al., 2021). This indicates that across such a long period of time, the injury caused by smoking may continue to progress or transform into IPF.

### 4.3 Body Aging and Cell Senescence

According to epidemiological surveys, advanced age is one of the most relevant demographic factors for IPF (Sgalla et al., 2018). The incidence of IPF in elderly men over 50 years of age is significantly higher than that in younger men (Raghu et al., 2018). However, the mechanism of how senescence leads to IPF is still unclear. In a mouse model of fibrosis, researchers observed a series of signs of cellular senescence, including elevated senescence-associated β-galactosidase (SA-β-Gal) in lysosomes and elevated reactive oxygen species (ROS) in mitochondria, an increase in cell cycle arrest proteins such as P53/P21/P16, an increase in the apoptosis-related protein BCL-2/Bax ratio, and an increase in DNA damage in the nucleus (Blokland et al., 2020). In addition, a variety of senescence-associated secretory phenotypes (SASPs) have been observed in animal models of pulmonary fibrosis, and these SASP components are known to include powerful profibrotic molecules such as TGF-β (Cho and Stout-Delgado, 2020; Rana et al., 2020). Similarly, studies have found that the lung fibroblasts of elderly IPF patients exhibit the above characteristics (Álvarez et al., 2017). Therefore, although there is a lack of human research evidence that senescence directly causes IPF, epidemiological data from both animal experiments and cell experiments suggest that IPF is a senescence-related disease.

The main manifestations of lung senescence are decreased lung tissue elasticity, decreased effective lung volume, the thickening of intercellular substances, and decreased lung function. Cell senescence mainly manifests as cell morphology and structure disorder, cell dysfunction, and cell growth/proliferation arrest (Rana et al., 2020).

The mechanism of pulmonary fibrosis caused by aging is not very clear, but there are quite a few theories to explain the mechanism of senescence in IPF. Cell senescence can disrupt of a variety of processes and generate imbalances (for example, shortened telomeres and DNA damage, oncogene activation, redox imbalances, mitochondrial dysfunction, lysosomal and proteasome dysfunction, ER stress), resulting in increased SASP, myofibroblast resistance to apoptosis, and stem cell depletion, ultimately manifesting as abnormal repair at the lung injury site (e.g., failure of the alveolar epithelium to regenerate properly and ECM deposition) (Sgalla et al., 2018; Glass et al., 2020; Liu and Liu, 2020; Merkt et al., 2020). Aging has been reported to reprogram the hematopoietic-vascular niche to impede regeneration and promote fibrosis (Chen et al., 2021).

Because senescent cells that resist apoptosis can continuously produce profibrotic cytokines, which in turn aggravate pulmonary fibrosis, the induction of apoptosis in senescent cells (e.g., dasatinib/quercetin, ABT-263, and NOX4 inhibitors) or selective antagonism of profibrotic senescence-related factors (e.g., IL-6, TGF-β or leukotrienes) may help to alleviate IPF (Merkt et al., 2020). Many antiaging drugs have passed preclinical animal studies and entered clinical trials. For example, dasatinib (D) and quercetin (Q) can promote the apoptosis of senescent cells. The DQ combination regimen has been investigated in a phase I clinical trial (NCT02874989). A total of 14 patients were enrolled in the trial; except for one patient who had severe adverse reactions (bacterial pneumonia), the remaining patients tolerated the treatment well, and improvements in parameters such as 6-min walking distance, 4-m gait speed, and chair stand time were reported (Schafer et al., 2017; Justice et al., 2019). Interestingly, Nifurtimox (NIF), originally used to treat diarrhea, has been reported to improve pulmonary fibrosis by blocking the production of myofibroblasts (Gan et al., 2022).

## 5 Recent Progress in Therapeutic Targets and New Drug Development for IPF

Pirfenidone and nintedanib are two drugs approved by the FDA for the treatment of IPF. They can delay lung function deterioration and prolong patient survival in IPF patients. Although the launch of Pirfenidone and nintedanib has demonstrated the feasibility of drug treatment for IPF, they do not reverse pulmonary fibrosis and their efficacy in patients with end-stage IPF is not fully clear. Therefore, there is a need to develop additional anti-pulmonary fibrosis drugs. The development of drugs for some specific targets is currently underway, which will be described in the following sections. In addition, drug combinations for antifibrotic therapy are worth investigating. Immune checkpoint inhibitors (ICIs), either as single agents or in combination regimens, have shown great success in clinical treatment (Wu et al., 2022). A case reports indicates that the addition of nintedanib to ICIs therapy might prevent drug-induced pneumonitis or acute exacerbation of IPF (Yamakawa et al., 2019).

### 5.1 Summary of Emerging Therapeutic Targets for IPF in Drug Discovery

In this section we briefly describe the current therapeutic targets in clinical trials and the latest clinical trial developments. We summarize the emerging therapeutic targets and representative mechanisms in the development of IPF (Table 4).

**TABLE 4:** Summary of emerging therapeutic targets for IPF in drug discovery.

Therapeutic targets	Mechanism	Ref
TGF-β	TGF-β is a powerful pro-fibrotic mediator	Ong et al. (2021)
αvβ6 Integrin	Integrin αvβ6 binds to an arginine-glycine-aspartic (RGD) sequence on LAP to activate latent form TGF-β1	Munger et al. (1999)
CTGF/CNN2	As a co-regulator of TGF-β in the pulmonary fibrosis microenvironment, CTGF can cooperate with TGF-β to participate in abnormal tissue repair processes including ECM production, fibroblast activation and differentiation	Lipson et al. (2012)
Galectin-3	Modulate macrophage phenotype/Gal-3 expression and fibroblast activation, reduce the effects of key profibrotic growth factors that act on myofibroblasts, and inhibiting EMT.	Humphries et al. (2021)
Leukotrienes	Leukotrienes have profibrotic effects by inducing fibroblast migration, proliferation, and matrix protein synthesis	Antoniou et al. (2007)
ATX-LPA-LPAR	The binding of LPA-to-LPAR can promote apoptosis of epithelial cells, regulation of endothelial permeability, activation of αvβ6 integrin-mediated TGF-β signaling, secretion of IL-8, recruitment and survival of fibroblasts. ATX is the key enzyme for LPA synthesis	Tager et al. (2008); Ninou et al. (2018); Suryadevara et al. (2020)
SPHK1-S1P-S1PR	The binding of S1P-to-S1PR can lead to mitochondrial reactive oxygen species (mtROS) and promote YAP1 to enter cell nuclei, affecting the differentiation of myofibroblasts and matrix remodeling. SPHK1 is the key enzyme for S1P synthesis	Huang et al. (2020)
PTX-2/SAP	As a ligand for the Fcγ receptor, PTX-2 downregulates monocyte and macrophage activity (especially M2)	Castaño et al. (2009)
JAK	JAK/STAT is a downstream pathway of IL-6, IL-11, IL-13, PDGF, TGF-β1 and FGF. The effect of JAK/STAT phosphorylation on cellular fibrotic processes includes proliferation, senescence, autophagy, endoplasmic reticulum stress, or epithelial/fibroblast to mesenchymal transition	Montero et al. (2021)
Src	Src is a group of nonreceptor tyrosine kinases, which participate in the TGF-β pathway by activating FAK.	Hu et al. (2014)
PI3K/Akt/mTOR	PI3K/Akt/mTOR plays a critical role in cell survival, growth, proliferation, protein synthesis, and EMT. *In vitro*, mTOR inhibitors can reduce TGF-β-induced fibroblast proliferation and type I collagen synthesis	Hennessy et al. (2005); Mercer et al. (2016); Lawrence and Nho, (2018)
Smo receptor	Smo is an important mediator of hedgehog signaling which is reactivated in adulthood within IPF	Effendi and Nagano, (2021)
Nitric oxide synthase	Activated macrophages, contributing to the cellular injury mediated by ROS, produce both nitric oxide (NO) and peroxynitrite	Giri, (2003)
GPR40/GPR84	GPR40 and GPR84 are G protein coupled receptors with free fatty acid ligands and are associated with metabolic and inflammatory disorders. PR40 agonist and GPR84 antagonists act on cells that involved in fibrotic pathways: macrophages, fibroblasts, and epithelial cells, and finally reduce inflammation	Gagnon et al. (2018)
LOX and LOXL	Lysyl oxidase (LOX) and LOX-like (LOXL) are enzymes involved in collagen cross-linking	Chen et al. (2019)

TGF-β plays an important role in the pathogenesis of IPF by stimulating the activation and proliferation of fibroblasts (Ong et al., 2021). Both anti-TGF-β mAb and anti-TGF-β mRNA nucleic acid have entered clinical trials (NCT00125385 and NCT03727802, respectively). Monoclonal antibodies against integrins that activate latent TGF-β have also entered phase II clinical trials (NCT04072315 and NCT04396756). Tyrosine kinase inhibitors targeting the TGF-β receptor have also entered phase II clinical trials (NCT03832946). Other growth factors (PDGF, VEGF, FGF, EGF, CTGF) are also involved in the process of pulmonary fibrosis. The mAb targeting CTGF, Pamrevlumab, demonstrated clinical benefit in phase II clinical trials and is now in phase III clinical trials (NCT03955146 and NCT04419558).

Chemokines and interleukins are also involved in recruitment of pro-fibrotic cells and formation of pro-fibrotic microenvironment, as we discussed in our previous paper (Ma et al., 2022). However, CCL2, a key chemokine for monocyte/macrophage migration and infiltration, failed to demonstrate clinical benefit in a phase II clinical trial (NCT00786201). Anti-IL-13 therapy didn’t contribute to lung function in patients with IPF (NCT02345070 and NCT01872689) or was terminated due to Lack of evidence of efficacy (NCT01266135 and NCT01266135).

Leukotrienes have profibrotic effects by inducing fibroblast migration, proliferation, and matrix protein synthesis (Antoniou et al., 2007). MN-001/Tipelukast, leukotriene receptor antagonist, is undergoing phase II clinical trial (NCT02503657).

As lipid proinflammatory mediators, The ATX-LPA-LPAR axis and SPHK1-S1P-S1PR axis have also been reported as targets of IPF. LPA and S1P have been demonstrated to promote the development of fibrosis (Tager et al., 2008; Ninou et al., 2018; Huang et al., 2020; Suryadevara et al., 2020). BMS-986278 (LPA1R antagonist) has entered phase II clinical trials (NCT04308681).

PTX-2, a ligand for the Fcγ receptor, can downregulate monocyte and macrophage activity (especially M2) (Castaño et al., 2009). PRM-151, a recombinant human pentraxin-2, has entered phase II clinical trials (NCT04594707 and NCT04552899).

Multiple cellular signaling pathways play a crucial role in the development of IPF. JAK/STAT signaling (Montero et al., 2021), receptor-type tyrosine kinase/non-receptor-type tyrosine kinase signaling (e.g., Src) (Hu et al., 2014), PI3K/Akt/mTOR signaling (Hennessy et al., 2005; Mercer et al., 2016; Lawrence and Nho, 2018), and Hedgehog signaling (Effendi and Nagano, 2021) comprise complex fibrosis regulatory signaling pathway. In our previous reviews, we have discussed the aforementioned signaling pathways (Ma et al., 2022).

There are also drugs in clinical trials targeting oxidative stress (Giri, 2003), GPR40/GPR84 (Gagnon et al., 2018), and collagen cross-linking enzymes (Chen et al., 2019).

### 5.2 Summary of Ongoing Clinical Trials for IPF

We searched clinical trials in recent years and summarized the anti-IPF drugs undergoing clinical trials and their mechanisms of action (Table 5). We also update the latest clinical trial progress for these drugs.

**TABLE 5:** Ongoing clinical trials about some investigational compounds for IPF.

Targets	Drugs	Clinical trial information
LPC-ATX-LPA	BMS-986278 (LPA1R antagonist)	Phase 2 (recruiting, NCT04308681)
PTX-2/SAP	PRM-151(Intravenous recombinant human pentraxin-2)	Phase 3 (recruiting, NCT04594707, NCT04552899)
CTGF/CNN2	FG-3019/Pamrevlumab (CTGF mAb)	Phase 3 (recruiting, NCT04419558, NCT03955146)
Galectin 3	TD139 (small-molecule antagonist of Galectin-3)	Phase 2 (recruiting, NCT03832946)
Oxidative stress	Niacin (nicotinic acid)	Phase 2 (recruiting, NCT0386592)
	Setanaxib/GKT137831(NOS1/4 inhibitor)	Phase 2 (recruiting, NCT03865927)
JNK	Jaktinib Dihydrochloride Monohydrate (JNK1/2 inhibitor)	Phase 2 (recruiting, NCT04312594)
	CC-90001 (JNK1/2 inhibitor)	Phase 2 (active, not recruiting, NCT03142191)
Src	Saracatinib (Src kinase inhibitor)	Phase 1/2 (recruiting, NCT04598919)
Hedgehog pathways	taladegib/ENV-101(Smo receptor inhibitor)	Phase 2 (not yet recruiting, NCT04968574)
Leukotrienes	MN-001/Tipelukast (leukotriene receptor antagonist)	Phase 2(Active, not recruiting, NCT02503657)
LOXL2	EGCG (irreversible inhibitor of both LOXL2 and TGF-β receptors 1 and 2 kinase)	Early Phase 1 (recruiting, NCT03928847)
IRE1	ORIN1001(IRE1 inhibitor)	Phase 1 (recruiting, NCT04643769)
PDE4b	BI 1015550(PDE4b inhibitor)	Phase 2 (active, not recruiting, NCT04419506)
NDMA	NP-120/Ifenprodil (N-methyl-d-aspartate (NDMA) receptor glutamate receptor antagonist)	Phase 2 (recruiting, NCT04318704)
B cell	Ianalumab/VAY736(B-cell activating factor receptor mAb)	Phase 2 (active, not recruiting, NCT03287414)
	Rituximab (CD20 chimeric mAb)	Phase 2 (active, not recruiting, NCT01969409); Phase 2 (recruiting, NCT03584802); Phase 2 (recruiting, NCT03286556); Phase2 (recruiting, NCT03500731)
Traditional medicine	Jin-shui Huan-xian granule	Not Applicable (recruiting, NCT04187690)
	Fuzheng Huayu tablet	Phase 2 (recruiting, NCT04279197)

## 6 Conclusion and Outlook

The research on the pathogenesis of IPF has made considerable progress. After years of basic and clinical studies, the pathogenesis of IPF has changed from simple inflammation to abnormal epithelial-mesenchymal crosstalk and other common pathogenic mechanisms. Based on this pathological mechanism, many studies have systematically studied the key roles of fibrocytes/(myo)fibroblasts, epithelial/endothelial cells, and ECM in the pathogenesis of IPF. With the progresses in understanding the key role of genetics and epigenetics in IPF, researchers have found that an increasing number of genetic loci and their apparent modifications are related to the maintenance of lung homeostasis although the effects of gene mutations and epigenetics on IPF still need further study.

The advent of pirfenidone and nintedanib is undoubtedly a sensational event in the field of IPF treatment, but they also have some major limitations, such as insufficient curative effect and poor pharmacokinetic properties. In addition, combination therapy based on them needs to be further studied. The number of clinical trials for therapies targeting cytokines, growth factors, and their signaling pathways is increasing rapidly. Furthermore, drugs targeting MMPs, telomerase activity, and epigenetic modifications need to be further developed.

Although IPF is idiopathic by definition, this deadly disease may become less mysterious with the progress in the understanding of the pathogenesis of IPF. The number of observed therapeutic targets is increasing, as well as the number of new drugs entering clinical trials. For patients with IPF, these advances described in this review show that slowing the IPF progression, prolonging their lives, and improving their quality of life might be possible in the future.
